# The impact of delayed surgical intervention following high velocity maxillofacial injuries

**DOI:** 10.1038/s41598-021-80973-7

**Published:** 2021-01-14

**Authors:** Daniel Oren, Amiel A. Dror, Adeeb Zoabi, Adi Kasem, Lior Tzadok, Fares Kablan, Nicole G. Morozov, Enssaf Safory, Eyal Sela, Samer Srouji

**Affiliations:** 1 Oral and Maxillofacial Department, Galilee Medical Center, Nahariya, Israel; 2 Department of Otolaryngology, Head and Neck Surgery, Galilee Medical Center, Nahariya, Israel; 3 The Sackler Faculty of Medicine, Tel Aviv University, Tel Aviv, Israel; 4 The Azrieli Faculty of Medicine, Bar-Ilan University, Safed, Israel

**Keywords:** Musculoskeletal system, Bone, Fracture repair, Prognosis, Reconstruction

## Abstract

Our study compares the number of postoperative complications of Syrian patients admitted to the Galilee Medical Center (GMC) over a 5-year period (May 2013–May 2018) for treatment after initial high-velocity maxillofacial injuries sustained during the Syrian civil war. Specifically, we evaluated complication rates of patients arriving “early,” within 24 h, to the GMC versus those who arrived “late,” or 14–28 days following high-velocity maxillofacial injuries. Both groups of patients received definitive surgical treatment within 48 h of admission to our hospital with a total of 60 patients included in this study. The mean age was 26 ± 8 years (range: 9–50) and all except one were male. Postoperative complications in the early group were found to be significantly higher compared to the delayed arrival group (p = 0.006). We found that unintentionally delayed treatment may have contributed to a critical revascularization period resulting in improved healing and decreased postoperative morbidity and complications. We discuss potential mechanisms for complication rate variations, including critical vascularization periods. Our study may add to a growing body of work demonstrating the potential benefit of delayed surgical treatment for high-velocity maxillofacial injuries.

## Introduction

Weapon injuries are generally categorized as *low-velocity* or *high-velocity* depending on the speed of impact from the weapon. American literature designates high-velocity as an artillery speed between 2000 to 3000 feet per second (610–914 m/s), whereas studies from the United Kingdom designate weapons with speeds above 1100 feet per second (335 m/s) as high velocity. War injuries are commonly induced by high-velocity weapons such as machine guns, improvised explosive devices, and missiles^1^. High-velocity injuries are significantly less common than those inflicted by low velocities, leading to comparatively less research available as to the proper surgical management of injuries sustained from high-velocity weapons. Our hospital, Galilee Medical Center, has had unique opportunities to evaluate, treat, and study these high-velocity weapons injuries sustained by hundreds of the 3200 Syrian patients who have arrived to our hospital between 2013 and 2018 in the midst of the Syrian civil war^2^.

Previous research has been conducted on maxillofacial injuries incurred during wars in Iraq, Afghanistan, and Israel by high-velocity weaponry. Prior studies highlight two primary treatment approaches: either rapid and immediate treatment of these injuries or staged and delayed treatment^3–6^. Many authorities advocate a conservative approach for the management of high-energy/high-velocity maxillofacial injuries which consists of, in order, debridement, fracture stabilization, and primary closure, followed by reconstruction of hard tissues and subsequently by correction of residual deformities of the oral cavity^3,4,7–13^.

Others advocate for the aforementioned procedures to be completed in one stage, rather than in many stages, as well as for the open treatment of all involved structures, which is greatly facilitated by the utilization of composite-free tissue transfer that provides intraoral lining, skin cover, and bone support in one step^5, 14–21^.

The vascularization of wounded tissue bears a marked impact on this critical revascularization period (CRP), or the time during which blood vessels at the site of injury self-repair, thus improving blood supply to the injured tissue^22^. Tissue regeneration is a complex process that includes numerous biological mechanisms, among them the essential process of angiogenesis^23,24^. In a mouse arteriovenous loop experimental study, very little angiogenesis or tissue generation occurs at early time-points (between 4 to 7 days) but these repairing mechanisms increase dramatically by 14 days and approximately double between day 14 and day 28^25^. It has been suggested that anastomoses should not be attempted until at least 2 weeks have elapsed from time of injury^5^. Based on the existing research and suggested timed delay of about 2 weeks for allowance of a CRP, we chose to compare the surgical results of patients treated immediately after injury and those inevitably treated 14–28 days after injury.

While the timing of intervention and the extent to which each step of treatment is applied may be controversial, we believe that allowing sufficient time for CRP, as suggested by delayed-treatment advocates, is an imperative step for providing optimal treatment outcomes. The purpose of this study is to compare the peri- and postoperative complication rates of early versus delayed treatment of high-velocity weapons injuries and to present the experience, strategies, and approach of our level I trauma center in treating severe facial injuries caused by high-velocity weapons.

## Methods

Informed consent was obtained from all participants prior to surgery or any surgical intervention. This retrospective case series study (NHR 0166-17) was approved by the Galilee Medical Center Helsinki Committee Institutional Review Board (IRB) in compliance with the public health regulations and provisions of the current harmonized international guidelines for good clinical practice (ICH-GCP) and in accordance with Helsinki principles. The Galilee Medical Center (GMC) Institutional Review Committee applies the principles of the Declaration of Helsinki and acts on the instructions of the Ministry of Health and procedure of internationally agreed upon, current, appropriate clinical procedures (ICH-GCP). Informed consent was obtained from all subjects for this retrospective study.

The medical files of all injured Syrian patients treated at GMC from May 2013 through May 2018 were reviewed. Those maxillofacial injuries classified as high-velocity injuries, e.g., gunshot wounds (GSW) from rifles and machine guns, missile injuries, and improvised explosive device (IED) explosions, were identified.

Data extracted from medical records included age; sex; mechanism, location, and type of injury; interval from injury to admission to GMC; Glasgow Coma Scale (GCS) on arrival; tracheostomy presence; intensive care unit (ICU) admission; and length of ICU stay. Also recorded were date, number, and type of OMFS surgeries performed; complications such as infection, rejection of hardware, bone or soft tissue grafts, oroantral fistula formation; excessive scarring; additional surgeries performed in other departments; and the dates of hospital admission and discharge. Some patients were first admitted to other departments (e.g., orthopedics, neurosurgery intensive care unit) and then transferred to the Oral and Maxillofacial Surgery and Department of Otolaryngology, Head and Neck Surgery departments, or vice versa. Patient medical records lacking any of the aforementioned information regarding mechanism and time of injury or containing insufficient data concerning treatment follow-up and/or complications were excluded from our study. Patients who arrived between 1 day after injury and 13 days after injury were treated according to our protocol outlined below but were excluded from our analyses for the purposes of statistical accuracy.

Due to the nature of the presented injuries and the fact that the study population consisted of Syrian nationals, patients could not be invited to return for follow-up care in outpatient clinics. Thus, the length of hospital stay represents the total time each patient spent not only in the OMFS department, but at GMC in total. Patient confidentiality was ensured by collecting only non-identifying information such as age, sex, injury classification, and Syrian national status. Identifying information such as name, identification data, and Syrian address were excluded for the purposes of this study and for each patient’s safety.

**Figure 1:**
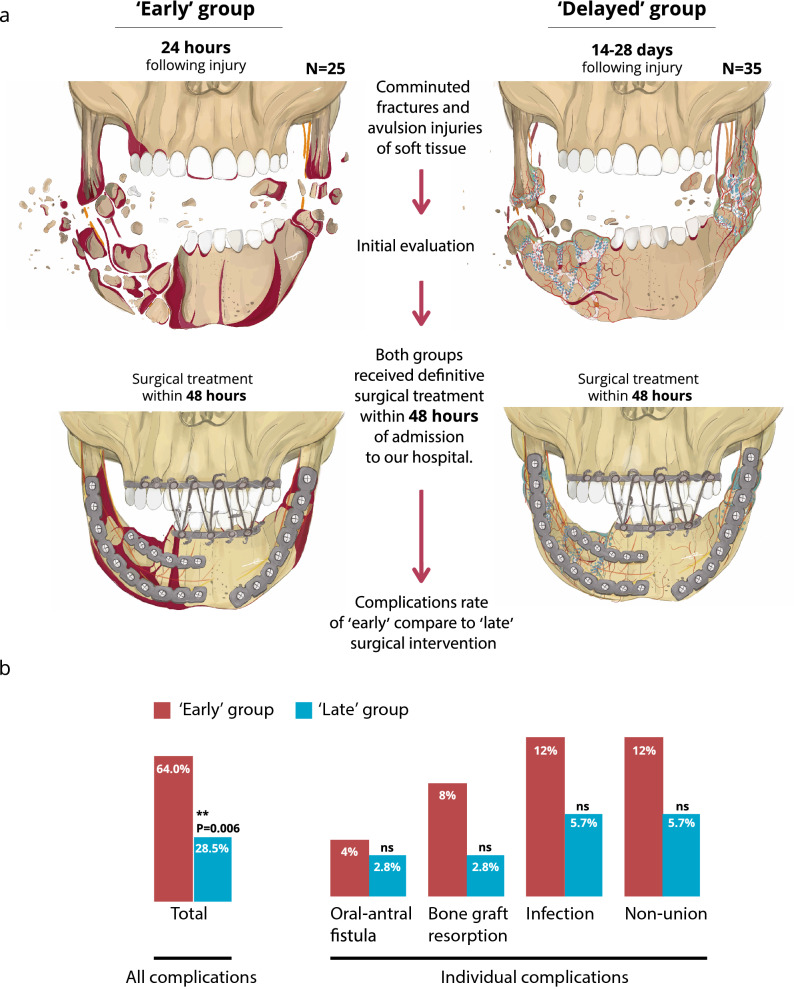
Patient timing of arrival at hospital following “high-velocity” maxillofacial injury and associated rate of post-operative complications. Image courtesy of Enssaf Safory.

Patients treated by GMC between May 2013 and May 2018 were divided into two groups: the first group included patients who arrived to GMC within 24 h of the initial injury while the second group included patients who arrived to the GMC between 14 and 28 days following injury. This delayed intervention group included patients who had minimal on-site treatment; in other words, debridement of wounds and simplification of fractures with no prior fixation had been performed in Syria. Upon arrival at our hospital, patients of both the early and delayed groups followed the same treatment protocol. Open reduction and internal fixation (ORIF) were conducted, bone defects were reconstructed with grafts, and soft tissue was covered with local flaps at GMC within 48 h of admission (Fig. 1a). All patients upon arrival were treated with tetanus prophylaxis and, throughout the course of their stays in the Department of Oral and Maxillofacial Surgery or Otolaryngology Head and Neck Surgery, received the same antibiotic protocol. Fifty-five were treated three times per day with 1 g of Augmentin (GSK-ISRAEL, LTD) while five patients allergic to penicillin were treated three times per day with 600 mg of clindamycin (Pfizer ISRAEL, LTD). In addition, all patients received the same postoperative treatment including intravenous (IV) fluid support, steroids (dexamethasone), and analgesic drug therapy.

The rates of individual complications, including soft tissue dehiscence and plate or bone exposure, oral-antral fistula, bone graft resorption, infection and non-union of fractures, and numbers of total complications among the delayed versus early intervention groups were analyzed. Each group was further divided into two subgroups according to the site of injury: lower face (mandible) or mid-upper face (maxilla, zygoma, orbits). Analysis of both subgroups combined was also performed.

Statistical analysis was performed with IBM SPSS statistics, version 25^26^. Quantitative data are described by mean, standard deviation, and range, while qualitative data are described by frequencies and percentages. Quantitative data between the early and delayed groups were compared using independent sample t-test while the qualitative data were compared using Pearson Chi-square test or Fisher's exact test (if expectancy < 5); differences between the two groups were considered significant at p-value < 0.05. Two-sided p-value analyses were also performed.

### Ethics approval and consent to participate

Ethical approval was acquired for this article.

## Results

Over 3200 Syrian patients were admitted and treated at GMC during the study period, 70% of whom presented with war-related injuries. Head and neck injuries were recorded for 350 patients (11%), 105 (30%) of whom suffered high-velocity, battlefield maxillofacial (MF) injuries. Of these patients, 45 were excluded due to lack of follow-up data. All 60 patients included in this analysis were treated by the same team of surgeons at GMC's Department of Oral and Maxillofacial Surgery or Department of Otolaryngology, Head and Neck Surgery. No statistical differences in demographics were noted between the early and delayed groups. The patients' mean age was 26 ± 8 years (range: 9–50) and all except one were male. Mean hospitalization duration at GMC was 73 ± 16 days (Table 1). Penetrating injuries were present in 37 patients (62%) while 15 (29%) suffered from perforating injuries. In addition, avulsive injuries were observed in 32 cases (53%). The specific clinical diagnoses were determined based on presentation, physical examination, and imaging. The distribution of each facial fracture diagnosis according to location of injury was as follows: 40 mandibular fractures (39%), 23 maxillary fractures (22%), 19 zygomatic fractures (18%), 18 orbital fractures (17%), and two Lefort III-type fractures (2%). Of the 40 mandibular fractures, 27 involved more than one fracture site per patient, totaling to 67 mandibular fractures. Of these 67 mandibular fractures, 26 were in the mandible body (39%), 19 were angular fractures (29%), 14 were of the symphysis (20%), seven were condylar/subcondylar (10%), and one involved coronoid fractures (2%). Thirty-four of the mandibular fractures were open to the skin, oral cavity, or both (85%). Thirty-one of the mandibular fractures were comminuted (77%). The early group included 25 (42%) patients while 35 (58%) were treated following delayed arrival to our center (Fig. 1).

**Table 1:** Patient demographics, baseline characteristics, and treatment course.

	Early group	Delayed group	Total	P value
Number of patients	25	35	60	
**Site of injury**				
Lower face	15 (60%)	25 (71.4%)	40 (60%)	0.412*
Comminuted	10 (40%)	21 (60%)	31 (51.7%)	0.190*
Mandible body	10 (40%)	16 (45.7%)	26 (43.3%)	0.793*
Angle	7 (28%)	12 (34.3%)	19 (31.7%)	0.779*
Symphysis	5 (20%)	9 (25.7%)	14 (23.3%)	0.760*
Condyle/subcondylar	3 (12%)	4 (11.4%)	7 (11.7%)	1.000**
Coronoid	0 (0%)	1 (2.9%)	1 (1.7%)	1.000**
Mid/upper face	14 (56%)	20 (57.1%)	34 (56.7%)	1.000*
Maxilla	9 (36%)	14 (40%)	23 (38.3%)	0.794*
Zygoma	7 (28%)	12 (34.3%)	19 (31.7%)	0.779*
Orbit	7 (28%)	11 (31.4%)	18 (30%)	1.000*
Lower/mid/upper face combination	4 (16%)	10 (28.6%)	14 (23.3%)	0.357*
Age (years), mean ± std	25 ± 9	26 ± 7	26 ± 8	0.645***
Gender	M(24) (96%) F(1)	M(35) (100%) F(0)	M(59) (98.3%) F(1)	0.417**
Interval from injury to admission, mean ± std	16 ± 10 (hours)	22 ± 8 (days)	Not applicable	0.105***
Preoperative time from admission to debridement/simplification (h), mean ± std	2 ± 3	3 ± 3	3 ± 3	0.208***
Additional surgeries (not maxillofacial)	23 (92%)	32 (91.4%)	55(91.7%)	1.000*
Mean hospitalization duration in GMC (days), mean ± std	70 ± 15	74 ± 17	73 ± 16	0.340***

*Chi square test; **Fisher's exact test; ***Independent sample t-test.

Complications included soft tissue dehiscence and plate or bone exposure, oral-antral fistula, bone graft resorption, infection, and non-union of fractures (Fig. 1b). Sixteen patients (64%) in the early group suffered from complications, while only ten patients (28.5%) in the delayed group suffered complications. When the incidence of each type of complication was compared separately, it was found that the delayed surgical intervention group had fewer complications than the early treated group. Application of the Pearson Chi-Square test to examine the complication rate for each group revealed that the total complication rate in the early treated group was significantly higher, statistically, compared to the delayed group (p value = 0.006) (Fig. 1b).

## Discussion

To date there is no consensus or well-established protocol for the treatment of maxillofacial high-velocity gunshot or artillery-related injuries despite the fact that the timing, sequence, and application of surgical procedures in reconstructing and rehabilitating maxillofacial injuries have been proven to influence the final outcome and aesthetic results^3,4,15,16^. However, high-energy transfers may result in temporary damage to the microcirculation of soft tissues distal to the permanent wound, which must be considered when planning microvascular anastomoses^27^.

Currently, cranio-maxillo-facial (CMF) trauma protocols are based primarily on the experience of treating soldiers injured in Iraq, Afghanistan, and Israel^5,28–37^. Early studies advocate the aggressive surgical treatment of facial gunshot injuries as early as possible to minimize scarring and contracture of facial soft tissues. This approach advises the early intervention of a multispecialty team integrating plastic surgical techniques, ORIF and miniplate reconstruction of facial fractures, acute bone grafting, and soft tissue reconstruction at the earliest possible opportunity^38^. In contrast, the staged, or delayed, treatment approach underscores the importance of staging hard and soft tissue treatment, which to a large degree depends on surgeon judgment, the extent of injury, and the general condition of the patient^3,4^.

Surgical management of maxillofacial wounds caused by high-velocity weapons generally consists of three stages: debridement, fracture stabilization and primary closure; followed first by reconstruction of hard tissues (provided that soft tissue coverage is adequate); then by rehabilitation of the oral vestibule, alveolar ridge and secondary correction of residual deformities^3,4,15,31,32^. Often, several operations may be required at any of the three stages. Selection of appropriate surgical technique and procedure is important, as improper technique may lead to infection, sequestration, wound dehiscence, graft rejection, or facial deformity, all of which may prolong hospital stay and postoperative morbidity as well as increase treatment costs^3,15^. Simple solitary facial fractures (without extensive soft tissue avulsion or infection) can be reduced, immobilized, and fixed at the time of primary closure using osteosynthesis plating in accordance with AO TRAUMA principles, provided that soft tissue coverage can be obtained and debridement is performed. As for all trauma injuries, bone fragments, especially those attached to the periosteum and muscle, must be located and reduced to ensure periosteal blood supply and tissue attachment during fragment redaction and hardware application. Leaving soft tissue defects open is best avoided since this may result in the extensive scarring of facial tissues and increased infection rates. In contaminated wound debridement, massive irrigation, and loose closure of the locally transferred tissue are required. Infection rates after war-related maxillofacial injuries have been poorly characterized, with rates ranging between 7 and 19%. All wounds from bullets or artillery fragments are inherently contaminated^39^.

From both the aesthetic and functional perspectives, local undermining and use of regional soft tissue advancement rotation flaps for primary closure of maxillofacial soft tissue defects have proven beneficial^3,4,15,16^. Hemodynamics must also be addressed, as oxygen-carrying capacities affect both wound healing and prevention of infection^40^. Here, the use of broad-spectrum antibiotic therapy, which provides aerobic and anaerobic coverage, plays a major role^3,4,15,16^.

In the current study, we focused mainly on the rates of complications, comparing early versus delayed treatments of high-velocity maxillofacial injuries. Sixty patients were treated for maxillofacial high-velocity battlefield injuries in accordance with AO TRAUMA principles. The overall complication rate was significantly higher in the early group than in the delayed group (p = 0.006), suggesting in our view the potentially important contributions of CRP to enhanced surgical outcomes.

## Conclusions

The Syrian civil war presented Oral, Maxillofacial, and Otolaryngology surgeons at the GMC with a unique and challenging opportunity to examine surgical outcomes of patients treated within 24 h of injury or between 14 and 28 days after injury. Our results, demonstrating a statistically significant higher surgical complication rate among patients treated early following injury, highlighted a potential benefit of physiologic vascular healing prior to planned intervention. The strikingly extensive revascularization of injured tissues during the 2 to 4 week period before arrival to GMC may have been the foundation for successful surgical intervention in these groups of patients. Our study therefore can potentially substantiate the results of pre-existing research suggesting a benefit from staged or delayed surgical approaches^3,4^.

It is unfeasible to draw unequivocal conclusions concerning the ideal time for definitive surgical treatment, yet our research findings indicate that delayed treatment, characterized by an opportunity for maxillofacial revascularization, can enhance surgical outcomes while simultaneously decreasing postoperative morbidity and complications.

Further substantiation of delayed treatment protocols with evaluation of CRP as a potential corollary between tissue revascularization and wound healing may be provided by ongoing basic research that recapitulates the molecular mechanisms of injury and healing timelines in humans. Defining the ideal schedule for surgical intervention will greatly assist trauma surgeons in providing optimal patient care.

## Data Availability

All data generated or analyzed during this study are included in this published article.

## Abbreviations

ATLS: Advanced trauma life support
CMF: Cranio-maxillo-facial
CRP: Critical revascularization period
GMC: Galilee Medical Center
MMF: Maxillo-mandibular fixation
ORIF: Open reduction internal fixation
